# Fast receptor-antigen dissociation kinetics promote CAR-T cell tumor infiltration, functional fitness, and antitumor efficacy in solid tumors

**DOI:** 10.64898/2026.10.10.756030

**Published:** 2026-10-12

**Authors:** Rui Mao, Huidong Shi, Mohamed S. Hussein, Bela Karia, Obed Koomson, Ramses Sadek, Vamsi Kota, Nicholas R.J. Gascoigne, Yukai He

## Abstract

**Background:**

CAR-T cells have shown limited efficacy against solid tumors, where they face two major challenges: restricted tumor infiltration and sustained intensive engagement that drive them to exhaustion. Conventional CAR design has largely emphasized high-affinity interactions with slow dissociation, further limiting tumor infiltration and exacerbating exhaustion and dysfunction. We hypothesize that most current CARs are kinetically over-stabilized for solid tumors and that a fast-on/fast-off, “ **cheek-kiss** ” mode of engagement permits transient CAR–antigen disengagement, thereby facilitating tumor infiltration and preserving CAR-T cell fitness in tumor mass.

**Methods:**

We identified two monoclonal antibodies targeting the same epitope of epidermal growth factor receptor with comparable *k* on but distinct *k* off rates: cetuximab (Cet) and its humanized derivative H1. CAR-T cells incorporating these binders were evaluated *in vitro* and *in vivo* . Large, established human tumor xenografts were treated with low-dose CAR-T cells in NSG mice to model conditions of limited T cell availability and sustained tumor antigen exposure.

**Results:**

Cet and H1 exhibited comparable *k* on but markedly different *k* off rates, with H1 displaying fast-off kinetics with approximately 18-fold shorter binding dwell time. As CARs, H1 showed reduced antigen occupancy and less stable tumor-T cell engagement than Cet. Despite comparable *in vitro* cytotoxicity, H1 CAR-T cells accumulated more efficiently following tumor stimulation. At high doses, both CAR-T cell products eradicated established tumors. Under low-dose conditions, however, H1 CAR-T cells exhibited superior expansion, persistence, tumor infiltration, and antitumor efficacy, whereas Cet CAR-T cells showed limited expansion and only transient tumor control. Cet CAR-T cells progressively downregulated surface CAR expression, increased PD-1 expression, and exhibited reduced tumor infiltration and impaired functional recovery. In contrast, H1 CAR-T cells preserved surface CAR expression, exhibited greater tumor infiltration, maintained a less exhausted, more central memory-like phenotype, and retained effector function.

**Conclusion:**

Fast-on/fast-off binding kinetics enhance CAR-T tumor infiltration, expansion, persistence, and functional fitness, resulting in improved control of established solid tumors. The data provides a rationale for shifting CAR design from affinity optimization toward kinetic optimization to improve the efficacy and safety of CAR-T therapy for solid tumors.

**WHAT IS ALREADY KNOWN ON THIS TOPIC:**

CAR design has largely focused on antigen-binding affinity, whereas the contribution of binding kinetics, particularly the dissociation rate, to CAR-T cell function, expansion, persistence, tumor infiltration, and antitumor efficacy remains poorly defined.

**WHAT THIS STUDY ADDS:**

In this study, we found that, compared with slow-off binding, fast-off kinetics enhance CAR-T cell tumor infiltration, expansion, persistence, and functional fitness in tumor-bearing mice, revealing previously underappreciated effects of antigen-binding kinetics on CAR-T cell tumor infiltration and performance in solid tumors.

**HOW THIS STUDY MIGHT AFFECT RESEARCH, PRACTICE OR POLICY:**

This study provides a rationale for incorporating antigen-binding kinetics into CAR design and optimization. The TCR-inspired fast-on/fast-off antigen engagement may help CAR T cells maintain fitness during sustained antigen exposure and thereby improve their infiltration, persistence, and antitumor activity in solid tumors.

